# Comprehensive Evaluation of Yunnan Potato Landraces: Agronomic, Sensory, and Nutritional Traits

**DOI:** 10.3390/foods14081298

**Published:** 2025-04-08

**Authors:** Ying Wang, Chunguang Yao, Jitian He, Lei Zhang, Jianming Bai, Yanshan Li, Jinhua Zhou, Beilei Zhao, Xianping Li, Zhechao Pan, Wanlin Yang

**Affiliations:** 1 Industrial Crops Research Institute, Potato Technology Innovation Center of Yunnan Province, Yunnan Academy of Agricultural Sciences, Kunming 650200, China; fengzheng1669@126.com (Y.W.); ycg@yaas.org.cn (C.Y.); zhanglei81@yaas.org.cn (L.Z.); bjm@yaas.org.cn (J.B.); lys@yaas.org.cn (Y.L.); zhoujh@yaas.org.cn (J.Z.); lixp@yaas.org.cn (X.L.); 2 Yunnan Provincial Academy of Food and Oil Sciences, Kunming 650033, China; kmhejitian@163.com; 3 Yunnan Popular Science Resource Information Center, Kunming 650021, China; qweasd202504@126.com

**Keywords:** China potato landraces, sensory characteristics, mineral elements, texture characteristics, starch pasting characteristics

## Abstract

This study focused on 48 landraces from Yunnan Province, with seven commercially bred varieties as controls. Descriptive analysis and variance analysis were employed to evaluate the landraces across various aspects, including agronomic traits, tuber characteristics, and quality attributes. The results indicated that compared with commercial varieties, landraces exhibited unique appearances with deeper eyes and smaller and irregularly shaped tubers. In terms of texture, landraces had greater firmness and poorer mealiness but possessed a richer potato flavor, resulting in higher overall scores than commercial varieties. The mineral content of landraces was slightly lower than that of commercial varieties but similar to that of Andean landraces. The starch from landraces demonstrated better thermal stability but was more prone to retrogradation. Textural properties showed that landraces had greater hardness, cohesiveness, springiness, and chewiness compared with commercial varieties, indicating a denser texture, poorer mealiness, greater firmness, and consistent results with the sensory evaluation. In summary, landraces represent crucial genetic resources in breeding programs. A comprehensive study of the sensory characteristics, agronomic traits, and mineral content of landraces is essential for effectively utilizing these resources to broaden the genetic base of potato germplasm.

## 1. Introduction

The term “landrace” was first mentioned at the International Agricultural and Forestry Congress held in Vienna in 1890 [1]. It is defined as a heterogeneous cultivated variety selected within specific ecological and geographical regions, which is well adapted to soil and climatic conditions, as well as traditional local management and usage practices. In China, potato landraces are primarily concentrated in Yunnan Province, Zhejiang Province, Tibet, and Sichuan Province. Cheng Linrun conducted mineral element testing on 24 landraces from Zhejiang Province and 22 cultivated varieties and found that the landraces did not have an advantage pertaining to the mineral element content [2]. Xu Juanni collected 15 landraces from Tibet and found that the starch content ranged from 15.35% to 21.55%, with all but three varieties having a starch content above 18% [3]. Gan Shengnan analyzed 18 landraces from Yunnan Province for traits such as branching, stem color, flower color, tuber shape, skin color, skin smoothness, flesh color, eye depth, plant late blight resistance, plant height, stem thickness, main stem number, and tuber size. The results showed a large genetic diversity index for these traits, indicating more significant diversity differences and higher richness [4]. Yang Xianquan et al. conducted RAPD analysis on 21 Sichuan potato landraces and 10 cultivated varieties, revealing potentially wider genetic variation among landraces with high breeding value [5].

**Figure 1:**
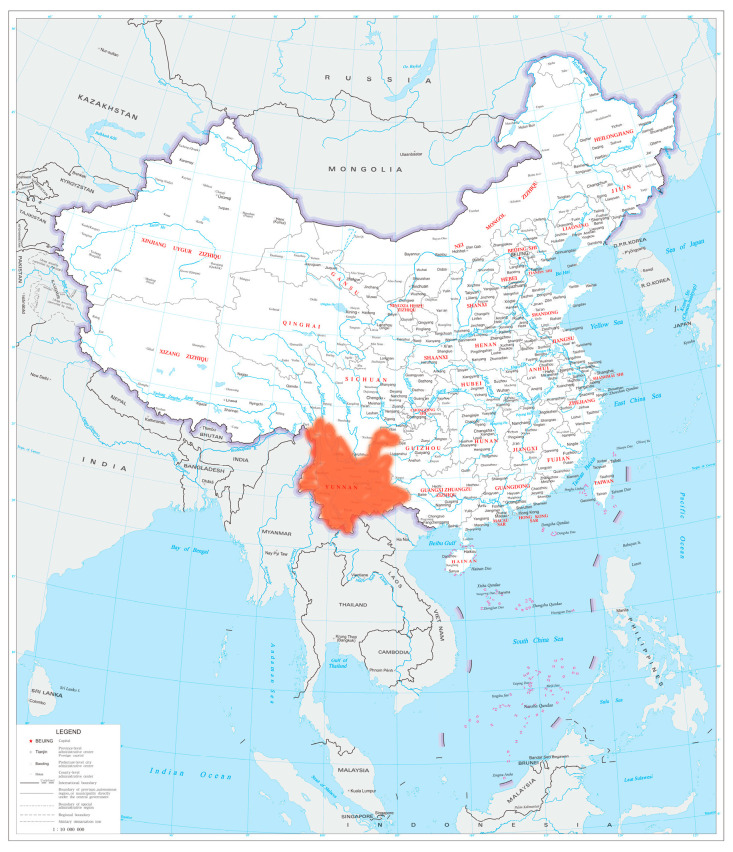
China’s map. Yunnan Province, located in the southwest of China, is marked in red on the map.

Yunnan is one of the earliest Chinese provinces to cultivate potatoes. According to records from Wu Qirui’s *Illustrated Examination of Plant Names*, which was written during the Qing Dynasty, different potato varieties were already present by 1848. These varieties, reportedly introduced by European missionaries who visited Yunnan Province, have been preserved over the years through local farmers’ selection and have become landraces [6]. Yunnan Province is located on the southwestern border of China (see Figure 1). Yunnan’s unique geographical conditions, especially its mountainous areas, possess latitudes, climates, and sunlight conditions similar to the Andes Mountains, where potatoes originated. Therefore, for over a hundred years, a large number of ancient and unique potato landraces have been preserved. In the Diqing Plateau of the Tibetan area, at an altitude of 3000 m, there are varieties like Zhong dian hong, Ni xi zi, and Ge zan bai. In the northwest Yunnan Plateau Bai area, at altitudes of 2000 to 2500 m, there are He qing hong and Jian chuan hong varieties. In the northeast Yunnan high mountain area, there are Zhuan xin wu, Yang ren yang yu, and Xuan wei ba ba yang yu varieties. In the Wenshan Zhuang Autonomous Prefecture, at an altitude of 1600 m, there is the Wen shan zi xin variety. In southern Yunnan, at around 1000 m, there are small waxy potatoes like Xiao nuo yang yu, Jing dong xiao yang yu, Xie di yang yu, and Wa ke yang yu among the Yi and Hani ethnic groups. In Kunming’s Dong Chuan district, there is a famous variety called Kai hua yang yu. These ethnically distinctive varieties are valuable germplasm resources [6]. In a preliminary study on landraces, a total of 292 global potato resources were subjected to genetic diversity and population structure analyses. This work found that potato landraces in Yunnan Province exhibited higher genetic diversity and greater genetic distance [7]. This indicates that landraces can serve as a potential gene pool for future potato-breeding programs to enrich China’s potato genetic pool. However, these landraces are scattered during planting, with unclear origins and disordered names. There is little research on the comprehensive evaluation of agronomic and quality traits of landraces, which hinders their development and utilization. Therefore, conducting a comprehensive evaluation of the traits of landraces and assigning values to them are crucial steps towards their sustainable development. This study evaluates the agronomic traits, tuber characteristics, tuber taste, tuber nutritional components, starch pasting properties, and tuber texture characteristics of landraces to gain a comprehensive understanding of them, laying the foundation for the further development and utilization of landraces. This study hypothesizes that Yunnan potato landraces exhibit unique agronomic and sensory traits compared to commercial varieties, offering valuable genetic resources for breeding programs aimed at improving diversity and consumer appeal.

## 2. Materials and Methods

### 2.1. Experimental Materials and Field Management

The experimental materials consisted of 48 potato landraces and 7 commercial varieties (see Table 1). The experiment was conducted in Yema Village, Huize County, Yunnan Province (103°22′26″ E, 26°06′17″ N, 2700 masl), during the 2018–2019 growing season. A randomized complete block design with three replications was used. Each plot covered an area of 16.8 m^2^, arranged in four rows per plot, with a row length of 6 m, a row spacing of 0.70 m, a plant spacing of 0.20 m, and an aisle width of 1.5 m. The quantity of base fertilizer was 975 kg/hectare of compound fertilizer; N:P:K = 15:15:15. After emergence, top dressing fertilizers were applied: urea at 300 kg/hectare and K_2_SO at 375 kg/hectare. Throughout the growing period, late blight was controlled 5 to 7 times.

### 2.2. Experimental Method

#### 2.2.1. Phenotypic Trait Measurement

During the potato growing period, flower color, plant type, and disease resistance were observed and recorded for each landrace. After harvesting the tubers, the skin color, flesh color, tuber shape, and eye depth were recorded, and the determination method used was the one proposed by Kathrin Thelen [8]. The field resistance of potato varieties was defined as the percentage of foliage with symptoms on a scale from 0% to 100%. This follows the severity characteristics utilized by Bock et al. [9].

#### 2.2.2. Quality Trait Measurement

(1) Determination of Mineral Element Content

The contents of phosphorus, zinc, iron, manganese, magnesium, copper, boron, calcium, and potassium were determined in accordance with NY/T1653-2008 [10].

(2) Starch Pasting Characteristics

The extraction protocol adhered to the methodology outlined by Slepkov et al. [11]. Potatoes were sectioned into small pieces and immersed in milli-Q water for a duration of 24 h at ambient temperature to facilitate starch extraction. Subsequently, the starch granules settled at the bottom of the beaker. The upper layer of the suspension was discarded, and the sediment was subjected to centrifugation at 3000 revolutions per minute (rpm) at 25 °C for 10 min. This procedure was repeated until a clear pellet was obtained, after which the supernatant was discarded. The gelatinization properties of potato starch were determined using a Rapid Visco Analyzer (RVA, Newport Scientific, Pty Ltd., Newcastle, Australia). In the RVA sample canister, starch (1.5 g, 12% d.b.) was mixed with 25 g of ultrapure water. The heating and cooling cycles were set as follows: (1) hold at 50 °C for 1 min, (2) heat to 95 °C over 3.8 min, (3) hold at 95 °C for 2.5 min, (4) cool to 50 °C over 3.8 min, and (5) hold at 50 °C for 1.4 min. During the first 10 s of the test, the RVA paddle speed was set at 960 revolutions per minute, after which the speed was reduced to 160 revolutions per minute [12].

(3) Tuber Textural Characteristics

The texture properties of steamed potato tubers were measured using a texture analyzer (TMS-Pro, FTC Co., Sterling, VA, USA). The parameters measured included hardness, cohesiveness, springiness, gumminess, and chewiness. The determination method was the one proposed by Li et al. [13]. The samples were subjected to compression testing under the TPA mode of the texture analyzer using a TMS 36.0 mm aluminum cylinder. The pre-test speed was set at 60 mm/min, the test speed at 30 mm/min, and the post-test speed at 60 mm/min. The trigger force was 0.7 N, with a waiting time between two compressions of 6 s and an indentation strain of 10%. After each test, the probe was wiped clean. Each sample within a group was tested in triplicate, and the mean value was taken as the final result, which was recorded.

(4) Sensory Evaluation

During the sensory evaluation, 10 expert panelists were involved in the assessment. Expert panelists possess specialized knowledge and experience in a particular sensory field. Experts contribute deep insights and nuanced feedback, which can be valuable in assessing premium or niche products [14].

The samples were randomly numbered, and the potatoes were steamed for 50 min before being longitudinally cut in half. The sensory evaluation form and the samples were then presented to the panelists. The panelists conducted their evaluations in separate tasting rooms without discussion. The overall sensory trait was evaluated based on five aspects: flavor, texture before tasting, initial texture upon tasting, chewing texture, and overall flavor [15]. The evaluation form is provided in Supplementary Materials Table S3.

### 2.3. Statistical Analysis

All data were the averages of three repetitions. Data processing was carried out using Origin 2021b for descriptive analysis, and the mean, standard deviation, minimum value, maximum value, and coefficient of variation of local and commercial varieties were analyzed. The ring chart, boxplots, and normal curve graphs were all drawn using Origin 2021b.

## 3. Results and Discussion

### 3.1. Investigation of Agronomic Traits of Landraces

The flower color, plant type, and disease resistance of landraces were investigated. The results are presented in the Supplementary Materials Table S1. The flower color of the potatoes included white, reddish purple, light pink, light purple, dark reddish purple, blue, purple blue, pink, and dark purple blue. Overall, 23 landraces had white flowers, accounting for 47.91%. A majority of potato plants were erect and loose. In Yunnan Province, since the main growing season for potatoes is from April to September every year when the rainfall is heavy, potato plants with a loose growth habit tend to lodge, resulting in poor ventilation between the leaves and leading to an outbreak of late blight. Therefore, for Yunnan Province, an erect type of potato plant is considered desirable. The varieties with better plant types include Ge zan hong and Dong chuan ma jiao gan. Most varieties have tall plants that are prone to lodging. The strongest disease resistance was observed in Zi lai yang yu, followed by Hao zi yang yu, Gong shan du yang yu, Lin cang yang yu, Tie chang hong, Xiao zi yang yu, He ba yang yu, Zao fen long, Ban na1 (red skin), He qing hong, Dong chuan she kuai yang yu, Dong chuan ma jiao gan, Xiao wu yang yu, Bai hua yang yu, Dong chuan mu duo, Long chuan hong pi, Nan dian hong, Zi hua yang yu, Lao jia yang yu (Xin ping), Yingjiang hong yu, Nu jiang xiao nuo yang yu, Yao zi yang yu, Liang he xiao hong yang yu, and Lan cang bendi yang yu. The disease-susceptible varieties were Hong da yan jing, Hong ma nan, and Hui ze hong. Overall, landraces exhibit strong disease resistance, but their plants generally tend to lodge easily.

### 3.2. Tuber Characteristics of Landraces

**Figure 2:**
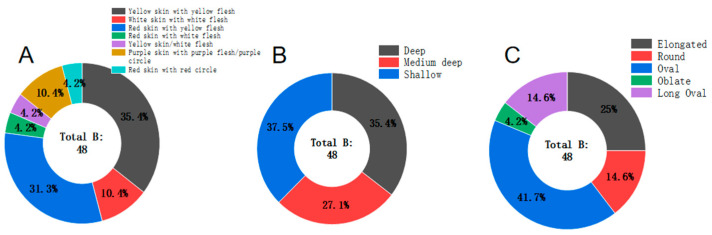
Distribution of potato skin and flesh color, eye depth, and tuber shape in landraces. (**A**): ring chart of landraces’ skin and flesh; (**B**): ring chart of landraces tuber eyes; (**C**): ring chart of landraces tuber shape.

**Figure 3:**
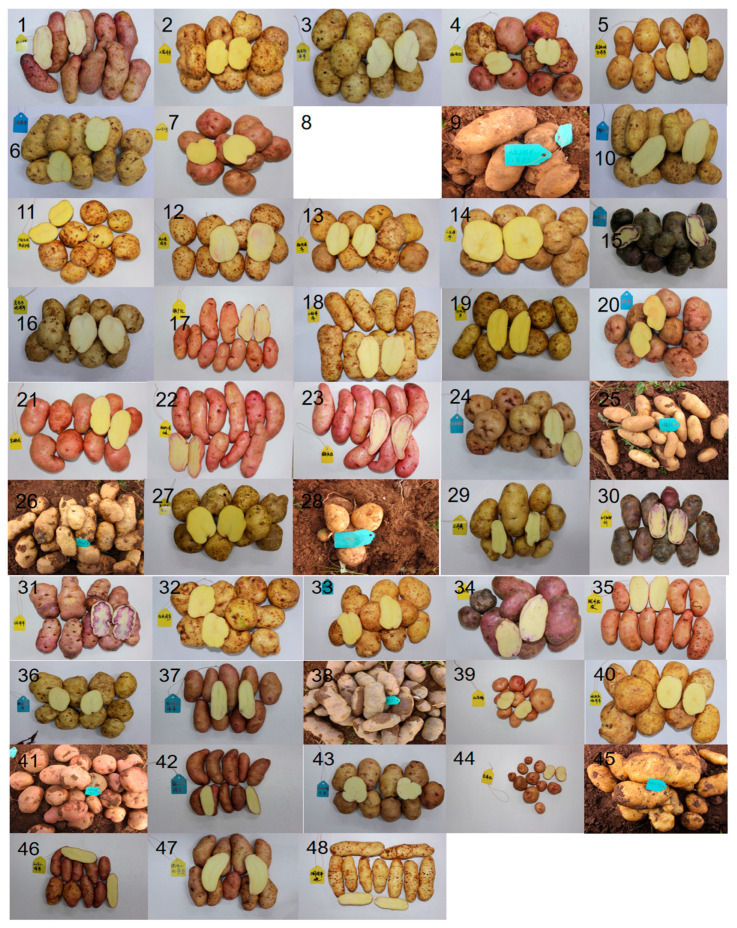
Skin, flesh, and shape traits of 48 potato landraces. 1: Haoziyangyu; 2: Dawoyangyu; 3: Luojibaiyangyu; 4: Gezanhong; 5: Hutiaoxiabaiyyangyu; 6: Wakeyangyu; 7: Gama2; 8: Tachenghongyangyu; 9: Banna1; 10: Guangnanbaipi; 11: Guangnanbaipi1; 12: Gongshanduyangyu; 13: Lincangyangyu; 14: Erwuyangyu; 15: Dongchuanxiaowuyangyu; 16: Jinggubendiyangyu; 17: Tiechanghong; 18: Xiaoziyangyu; 19: Hebayangyu; 20: Kaihuayangyu; 21: Zaofenlong; 22: Banna1 (hongpi); 23: Heqinghong; 24: Hongdayanjing; 25: Xiaonuoyang; 26: Xinluliang1; 27: Dongchuanshekuaiyangyu; 28: Chaomila; 29: Sanyuehuang; 30: Dongchuanmajiaogan; 31:Xiaowuyyangyu; 32: Zilaiyangyu; 33: Baihuayyangyu; 34:Dongchuanmuduo; 35: Longchuanhongpi; 36: Tiekeyangyu; 37: Nandianhong; 38: Zihuayangyu; 39: Hongmanan; 40: Weixiqingfuyangyu; 41:Laojiayangyu (Xinping); 42: Yingjianghongyu; 43: Nujiangxiaonuoyangyu; 44: Huizehong; 45: Yaoziyangyu; 46: Honghexiaoyangyu; 47: Lianghexiaohongyangyu; 48: Lancangbendiyangyu; 8 is “tacehnghongyangyu”, and the photos of this variety were not retained.

The tuber characteristics survey of landraces included skin color, flesh color, tuber shape, and eye depth. The results are presented in Supplementary Materials Table S2, Figure 2 and Figure 3. According to these results, the landraces with yellow skin and yellow flesh accounted for 35.4%, red skin with yellow flesh accounted for 31.3%, white skin with white flesh constituted 10.4%, and other types represented 23%. This indicates that yellow-skinned and yellow-fleshed varieties, along with red-skinned and yellow-fleshed ones, predominate among landraces, making up 66.7%. Varieties with medium deep to deep eyes accounted for 62.5%. Oval-shaped tubers comprised 41.7%, while long and elongated tubers made up 39.6%. This suggests that, compared to commercial varieties, landraces do not have an ideal appearance and tend to have more elongated or ovate tubers with deeper eyes.

### 3.3. Diversity Analysis of Sensory Characteristics in Landraces

**Figure 4:**
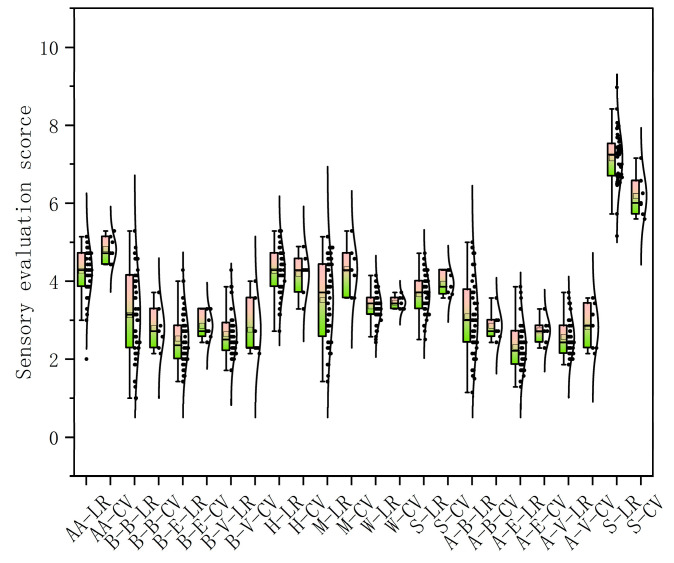
Boxplots and normal curves of sensory characteristics of landraces and commercial varieties. AA-LR: appearance acceptability of landraces; AA-CV: appearance acceptability of commercial varieties; B-B-LR: pre-tasting buttery flavor of landraces; B-B-CV: pre-tasting buttery flavor of commercial varieties; B-E-LR: pre-tasting earthy flavor of landraces; B-E-CV: pre-tasting earthy flavor of commercial varieties; B-V-LR: pre-tasting boiled vegetable flavor of landraces; B-V-CV: pre-tasting boiled vegetable flavor of commercial varieties; H-LR: hardness of landraces; H-CV: hardness of commercial varieties; M-LR: mealiness of landraces; M-CV: mealiness of commercial varieties; W-LR: waxiness of landraces; W-CV: waxiness of commercial varieties; S-LR: stickiness of landraces; S-CV: stickiness of commercial varieties; A-B-LR: post-tasting buttery flavor of landraces; A-B-CV: post-tasting buttery flavor of commercial varieties; A-E-LR: post-tasting earthy flavor of landraces; A-E-CV: post-tasting earthy flavor of commercial varieties; A-V-LR: post-tasting boiled vegetable flavor of landraces; A-V-CV: post-tasting boiled vegetable flavor of commercial varieties.

The results of the sensory characteristic diversity analysis of landraces are presented in Table 2 and Figure 4. Among the sensory characteristics of landraces, the pre-tasting buttery flavor had the highest coefficient of variation, with a mean value of 3.13. The lowest coefficient of variation was observed in the overall score, with a mean value of 7.15. In commercial varieties, the post-tasting cooked vegetable water flavor had the highest coefficient of variation with a mean value of 2.76. The lowest coefficient of variation was found for waxiness, with a mean value of 3.45. Compared with commercial varieties, landraces had lower acceptability in terms of appearance (2.46), poorer mealiness (3.51), and greater hardness (4.27) but excelled in flavor (pre-tasting buttery flavor: 3.13; post-tasting buttery flavor: 3.09), resulting in a higher overall score (7.15) than the commercial varieties (6.18). The flavor of potatoes directly impacts their sensory quality, which in turn influences consumer acceptance and purchasing decisions. Flavor is determined by a combination of various volatile and non-volatile compounds, including sugars, amino acids, fatty acids, and their derivatives [16]. Significant differences in flavor exist among different potato varieties, which are closely related to their genetic background and growth environment. Through breeding and genetic engineering techniques, it is possible to develop potato varieties with specific flavor profiles to meet diverse consumer demands [17].

This indicates that although landraces may be inferior to commercial varieties in appearance, such as tuber shape and eye depth, they have an advantage in terms of flavor. Breeding new potato varieties with higher sensory scores and greater nutritional value to meet the needs of consumers, producers, and processors should be the current focus of potato quality breeding [18].

### 3.4. Diversity Analysis of Mineral Elements in Potato Landraces

The results of the mineral element diversity analysis of landraces and commercial varieties in Yunnan Province in China are shown in Table 3. The iron content ranges from 3.8 to 35.45 mg/kg FW, zinc content from 1.68 to 3.61 mg/kg FW, calcium content from 30.40 to 69.70 mg/kg FW, phosphorus content from 340 to 671.5 mg/kg FW, magnesium content from 136.13 to 345.5 mg/kg FW, copper content from 0.59 to 4.20 mg/kg FW, boron content from 0 to 1.21 mg/kg FW, and manganese content from 1.07 to 2.99 mg/kg FW. The highest zinc content was found in Hong da yan jing, at 4.1 mg/kg, while the lowest was found in Banna 1 hongpi, at 1.69 mg/kg. The highest iron content was found in Lan cang ben di yang yu, at 35.45 mg/kg, while the lowest was found in Guang nan bai pi, at 3.8 mg/kg.

For commercial varieties, the iron content ranges from 8.58 to 37.20 mg/kg FW, zinc content from 1.68 to 3.61 mg/kg FW, calcium content from 36.95 to 61.25 mg/kg FW, phosphorus content from 396 to 711.5 mg/kg FW, magnesium content from 206.5 to 270.00 mg/kg FW, copper content from 0.56 to 3.66 mg/kg FW, boron content from 0.19 to 1.30 mg/kg FW, and manganese content from 1.46 to 1.89 mg/kg FW. Among the mineral elements in landraces, copper has the highest coefficient of variation, while magnesium has the lowest. In commercial varieties, iron has the highest coefficient of variation, and manganese has the lowest. Compared to commercial varieties, landraces have higher manganese and magnesium contents but slightly lower contents of zinc, phosphorus, iron, calcium, copper, and boron. Manganese is a trace mineral. The body requires manganese for immune function, blood sugar regulation, digestion, reproduction, bone health, blood clotting, antioxidant defense, and many more bodily processes [19]. Manganese toxicity primarily affects the central nervous system and can lead to symptoms such as tremors, hearing loss, muscle spasms, loss of appetite, mania, and headaches (National Institutes of Health: Office of Dietary Supplements. Manganese). Although there are no reports of manganese toxicity from dietary intake, the Food and Nutrition Board (FNB) has established a Tolerable Upper Intake Level (UL) for manganese, which is currently set at 11 milligrams per day for people aged 19 and older. Magnesium plays a role in over 300 enzyme reactions in the human body. Magnesium functions include helping muscle and nerve function, regulating blood pressure, and supporting the immune system. High levels of magnesium derived from food do not pose a health risk in healthy individuals because the kidneys eliminate excess amounts through urine [20]. However, high doses of magnesium from dietary supplements or medications often result in diarrhea that can be accompanied by nausea and abdominal cramping [21]. Therefore, although the content of manganese and magnesium in local varieties is higher than that in commercial varieties, consuming local varieties will not pose a hazard to the human body.

Andre et al. [22] reported that the iron content of Andean landraces ranged from 5.97 to 30.99 mg/kg FW, zinc content from 2.52 to 5.77 mg/kg FW, and calcium content from 54.38 to 218.59 mg/kg FW. Anderson et al. [20] published the iron content of unpeeled potatoes from the United States and Canada (2.34~26.21 mg/kg FW). The iron content in potatoes from Yunnan Province in China and in Peruvian potatoes is higher (16.08 mg/kg FW and 18.48 mg/kg FW, respectively) when compared to the iron content of unpeeled potatoes from the United States and Canada. This can be attributed to two factors: firstly, the similar terrain and altitude in Yunnan Province in China and Peru, combined with the predominantly acidic soils, are known to increase the availability of iron in the soil, thus potentially increasing the iron content in potato tubers [23]; secondly, genotype characteristics. Overall, the mineral nutritional value of landraces from Yunnan, China, is slightly lower than that of commercial varieties, although it is similar to that of Andean landraces.

### 3.5. Diversity Analysis of Starch Pasting Characteristics in Landraces

The starch pasting characteristics of landraces are shown in Table 4. The peak viscosity of landraces ranges from 9995 RVU to 14,607 RVU, with a coefficient of variation of 8.1%. The peak viscosity of landraces (12,368 RVU) is slightly higher than that of commercial varieties (12,354 RVU), though the difference is not significant. The breakdown value of starch in landraces ranges from 6678 RVU to 12,523 RVU, with a coefficient of variation of 11.84% and an average value of 10340 RVU, which is lower than that of commercial varieties (10,468 RVU), indicating that the starch of landraces exhibits better thermal stability. The setback value of starch in landraces ranges from 515 RVU to 3277 RVU, with a high coefficient of variation of 36.12%, indicating significant variability in the setback values of landraces’ starches, with an average value of 2102 RVU, which is higher than that of commercial varieties (2068 RVU). This suggests that landraces’ starches have a higher degree of retrogradation upon heating. The gelatinization temperature of landraces starch ranges from 62.6 °C to 70.3 °C, with a coefficient of variation of 2.85%, indicating minimal variation in the gelatinization temperatures of landraces’ starches, with an average value of 67.2 °C, which is slightly lower than that of commercial varieties (68.1 °C). However, these differences are not significant.

### 3.6. Diversity Analysis of Textural Characteristics in Landraces

Hardness refers to the maximum force required to break solid food when measured by an instrument or during sensory chewing. The hardness of landraces (630 N) is higher than that of commercial varieties (504 N). Both gumminess and chewiness reflect the energy required to bite and chew the sample into pieces. According to Table 5, landraces (101 and 59) have higher gumminess and chewiness compared to commercial varieties (90 and 43). Cohesiveness measures the relative resistance to the second compression after the first deformation, revealing the tightness of internal bonding within the tuber. Springiness indicates the ability of the potato tuber to recover its original shape after being compressed and then decompressed. The springiness of landraces is 0.60, higher than that of commercial varieties at 0.51. The analysis of textural characteristics shows that landraces have higher hardness, peak load, gumminess, springiness, and chewiness values compared to commercial varieties, indicating that landraces have a denser texture, poorer mealiness, and greater hardness, and these results are consistent with the conclusions of sensory evaluations.

## 4. Conclusions

Currently, commercial potato varieties are bred based on requirements such as smooth skin, ovate shape, and shallow eyes. Consequently, the market predominantly contains yellow-skinned, yellow-fleshed, ovate potatoes. This limited diversity in commercial potatoes may restrict consumer choice and nutritional options, whereas landraces offer a wider range of flavors and nutritional profiles. In contrast, landraces with unique shapes, rich colors, and good taste are favored by consumers, as evidenced by their higher comprehensive sensory scores compared with those of commercial varieties. Additionally, these colorful landraces are rich in anthocyanins and carotenoids, contributing to a more diverse nutritional profile for potatoes. Therefore, the development and utilization of these distinctive landraces with superior taste and attributing value to them are essential for the advancement of the potato industry.

The continuous advancement of society means that people’s demands for food are continuously increasing. Therefore, this implies that our breeding objectives need to be adjusted based on market demands. A high sensory score (good taste) is crucial for potato consumption, and developing new potato varieties with “good taste” increases consumer enthusiasm for potatoes [15]. The flavor (pre-tasting buttery flavor), taste (post-tasting buttery flavor), and overall sensory score of landraces are all higher than those of commercial varieties. Landraces have been proven to be useful in improving potato quality traits (flavor, texture, etc.). Therefore, fully exploring and utilizing the sensory genes of landraces may be the key breakthrough in potato quality breeding.

## Figures and Tables

**Table 1:** Information on potato landraces of Yunnan Province.

No.	Variety Name	No	Variety Name	No	Variety Name	No	Variety Name	No	Variety Name
1	Haoziyangyu	12	Gongshanduyangyu	23	Heqinghong	34	Dongchuanmuduo	45	Yaoziyangyu
2	Dawoyangyu	13	Lincangyangyu	24	Hongdayanjing	35	Longchuanhongpi	46	Honghexiaoyangyu
3	Luojibaiyangyu	14	Erwuyangyu	25	Xiaonuoyang	36	Tiekeyangyu	47	Lianghexiaohongyangyu
4	Gezanhong	15	Dongchuanxiaowuyangyu	26	Xinluliang1	37	Nandianhong	48	Lancangbendiyangyu
5	Hutiaoxiabaiyyangyu	16	Jinggubendiyangyu	27	Dongchuanshekuaiyangyu	38	Zihuayangyu	49	YS505
6	Wakeyangyu	17	Tiechanghong	28	Chaomila	39	Hongmanan	50	YS702
7	Gama2	18	Xiaoziyangyu	29	Sanyuehuang	40	Weixiqingfuyangyu	51	Cooperation88
8	Tachenghongyangyu	19	Hebayangyu	30	Dongchuanmajiaogan	41	Laojiayangyu(Xinping)	52	YS304
9	Banna1	20	Kaihuayangyu	31	Xiaowuyyangyu	42	Yingjianghongyu	53	Qingshu9
10	Guangnanbaipi	21	Zaofenlong	32	Zilaiyangyu	43	Nujiangxiaonuoyangyu	54	YS401
11	Guangnanbaipi1	22	Banna1(hongpi)	33	Baihuayyangyu	44	Huizehong	55	Yunxuan2

**Table 2:** Variations in sensory characteristics between landraces and commercial potato varieties.

Landraces					
	Mean	Standard Deviation	Minimum	Maximum	Coefficient of Variation/%
Appearance acceptability	4.26	0.61	2.00	5.14	14.39
Pre-tasting buttery flavor	3.13	1.10	1.00	5.29	35.27
Pre-tasting earthy flavor	2.52	0.68	1.43	4.29	26.99
Pre-tasting boiled vegetable flavor	2.64	0.56	1.71	4.29	21.20
Hardness	4.27	0.59	2.71	5.29	13.79
Mealiness	3.51	1.05	1.43	5.14	29.86
Waxiness	3.35	0.40	2.43	4.14	11.96
Stickiness	3.67	0.51	2.50	4.71	13.83
Post-tasting buttery flavor	3.09	1.03	1.14	5.00	33.42
Post-tasting earthy flavor	2.31	0.59	1.29	3.86	25.44
Post-tasting boiled vegetable flavor	2.57	0.48	1.86	3.71	18.63
Overall score	7.15	0.66	5.16	8.97	9.28
Commercial varieties					
Appearance acceptability	4.82	0.34	4.43	5.28	7.00
Pre-tasting buttery flavor	2.80	0.55	2.14	3.71	19.76
Pre-tasting earthy flavor	2.86	0.34	2.43	3.28	11.90
Pre-tasting boiled vegetable flavor	2.76	0.74	2.14	4.00	26.73
Hardness	4.19	0.53	3.29	4.89	12.71
Mealiness	4.31	0.62	3.57	5.28	14.39
Waxiness	3.45	0.17	3.29	3.71	5.03
Stickiness	3.93	0.30	3.57	4.28	7.69
Post-tasting buttery flavor	2.86	0.38	2.43	3.57	13.23
Post-tasting earthy flavor	2.73	0.32	2.29	3.28	11.85
Post-tasting boiled vegetable flavor	2.82	0.59	2.14	3.57	20.86
Overall score	6.18	0.54	5.59	7.16	8.75

**Table 3:** Variations in mineral contents between landraces and commercial varieties.

Landraces					
	Mean	Standard Deviation	Minimum	Maximum	Coefficient of Variation/%
Zinc (mg/kg)	2.48	0.48	1.69	4.10	19.36
Phosphorus (mg/kg)	501.05	92.04	340.00	671.50	18.37
Iron (mg/kg)	16.08	6.00	3.80	35.45	37.32
Manganese (mg/kg)	2.00	0.48	1.07	2.99	23.91
Magnesium (mg/kg)	237.55	37.70	136.13	345.50	15.87
Calcium (mg/kg)	43.92	9.24	30.40	69.70	21.04
Copper (mg/kg)	1.51	0.71	0.59	4.20	46.67
Boron (mg/kg)	0.71	0.26	0.00	1.21	37.02
Commercial varieties					
Zinc (mg/kg)	2.53	0.67	1.68	3.61	26.47
Phosphorus (mg/kg)	527.64	111.46	396.00	711.50	21.12
Iron (mg/kg)	16.85	9.55	8.58	37.20	56.64
Manganese (mg/kg)	1.76	0.14	1.46	1.89	7.94
Magnesium (mg/kg)	238.93	22.21	206.50	270.00	9.30
Calcium (mg/kg)	45.16	8.26	36.95	61.25	18.29
Copper (mg/kg)	1.58	0.97	0.56	3.66	61.26
Boron (mg/kg)	0.86	0.41	0.19	1.30	47.36

**Table 4:** Variations in starch pasting properties between landraces and commercial varieties.

Landraces					
	Mean	Standard Deviation	Minimum	Maximum	Coefficient of Variation/%
PV/RVU	12,368.27	1008.74	9995.00	14,607.00	8.16
TV/RVU	2027.56	887.26	895.00	4267.00	43.76
BD/RVU	10,340.71	1224.87	6678.00	12,523.00	11.85
FV/RVU	4130.02	418.11	3400.00	5348.00	10.12
SB/RVU	2102.46	759.52	515.00	3277.00	36.13
PT/°C	67.19	1.91	62.60	70.30	2.85
Commercial varieties					
PV/RVU	12,354.43	1056.37	11,467.00	14,286.00	8.55
TV/RVU	1886.43	310.52	1407.00	2424.00	16.46
BD/RVU	10,468.00	1036.95	9272.00	12,223.00	9.91
FV/RVU	3954.86	418.47	3384.00	4514.00	10.58
SB/RVU	2068.43	545.81	960.00	2644.00	26.39
PT/°C	68.10	0.44	67.70	68.70	0.65

PV: peak viscosity; TV: trough viscosity; BD: breakdown; FV: final viscosity; SB: setback; PT: pasting temperature.

**Table 5:** Variations in texture properties between landraces and commercial varieties.

Landraces					
	Mean	Standard Deviation	Minimum	Maximum	Coefficient of Variation/%
Hardness/N	630.96	229.67	178.28	1139.73	0.36
Peak load/N	734.00	252.22	281.63	1596.68	0.34
Springiness/mm	0.60	0.14	0.29	0.85	0.23
Cohesiveness/ratio	0.16	0.08	0.04	0.42	0.49
Gumminess/N	101.40	56.23	15.66	276.80	0.55
Chewiness/mj	58.72	35.54	11.04	183.32	0.61
Commercial varieties					
Hardness/N	504.86	180.76	201.19	762.37	0.36
Peak load/N	545.29	225.56	148.18	758.26	0.41
Springiness/mm	0.51	0.16	0.23	0.69	0.32
Cohesiveness/ratio	0.18	0.10	0.07	0.36	0.53
Gumminess/N	90.24	53.15	12.85	166.63	0.59
Chewiness/mj	43.50	26.80	3.24	93.85	0.62

## Data Availability

The original contributions presented in this study are included in the article/Supplementary Materials. Further inquiries can be directed to the corresponding authors.

## Supplementary Materials

The following supporting information can be downloaded at: https://www.mdpi.com/article/10.3390/foods14081298/s1, Table S1: Agricultural characteristics; Table S2: Tuber characteristics; Table S3: Sensory evaluation
